# Sex-dependent effects of amyloidosis on functional network hub topology is associated with downregulated neuronal gene signatures in the APPswe/PSEN1dE9 double transgenic mouse

**DOI:** 10.1101/2024.05.13.593982

**Published:** 2024-08-02

**Authors:** Zachary D Simon, Karen N McFarland, Todd E Golde, Paramita Chakrabarty, Marcelo Febo

## Abstract

Extracellular beta-amyloid (Aβ) is thought to cause impairments in brain-wide functional connectivity, although mechanisms linking Aβ to broader functional network processing remain elusive. In the present study, we evaluated the effects of Aβ on fear memory and functional connectome measures in male and female mice. Middle-aged (9-11mo) double transgenic APP-PS1 mice and age and sex-matched controls were tested on a fear conditioning protocol and then imaged at 11.1 Tesla. Brains were harvested and processed for analysis of Aβ plaques and Iba1 immunolabeling in neocortical areas, hippocampus, and basolateral amygdala. Additional RNA sequencing data from separate age, strain, and sex-matched mice were analyzed for differentially expressed genes (DEGs) and weighted gene co-expression networks. In both male and female mice, we observed increased functional connectivity in a dorsal striatal/amygdala network as a result of Aβ. Increased functional connectivity within this network was matched by increases in APP gene expression, Aβ and Iba1 immunolabeling, and an upregulated cluster of DEGs involved in the immune response. Conversely, the network measure representing node hubness, eigenvector centrality, was increased in prefrontal cortical brain regions, but only in female APP-PS1 mice. This female-specific effect of amyloid was associated with down-regulation of a cluster of DEGs involved in cortical and striatal GABA transmission, anxiogenic responses, and motor activity, in female APP-PS1 mice, but not males. Our results contribute to a growing literature linking between Aβ, immune activation, and functional network connectivity. Furthermore, our results reveal outcomes of Aβ on gene expression patterns in female mice that may contribute to amyloidosis-induced dysregulation of non-cognitive circuitry.

## Full Text Availability

The license terms selected by the author(s) for this preprint version do not permit archiving in PMC. The full text is available from the preprint server.

